# Characterizing cancer and COVID-19 outcomes using electronic health records

**DOI:** 10.1371/journal.pone.0267584

**Published:** 2022-05-04

**Authors:** Youngran Kim, Liang Zhu, Huili Zhu, Xiaojin Li, Yan Huang, Chunhui Gu, Heather Bush, Caroline Chung, Guo-Qiang Zhang

**Affiliations:** 1 Department of Neurology, McGovern Medical School, The University of Texas Health Science Center at Houston, Houston, TX, United States of America; 2 Hematology and Oncology Department, Baylor College of Medicine, Houston, Texas, United States of America; 3 Department of Biostatistics, College of Public Health, The University of Kentucky, Lexington, Kentucky, United States of America; 4 Department of Radiation Oncology, The University of Texas MD Anderson Cancer Center, Houston, Texas, United States of America; University of South Carolina, UNITED STATES

## Abstract

**Purpose:**

Patients with cancer often have compromised immune system which can lead to worse COVID-19 outcomes. The purpose of this study is to assess the association between COVID-19 outcomes and existing cancer-specific characteristics.

**Patients and methods:**

Patients aged 18 or older with laboratory-confirmed COVID-19 between June 1, 2020, and December 31, 2020, were identified (n = 314 004) from the Optum® de-identified COVID-19 Electronic Health Record (EHR) derived from more than 700 hospitals and 7000 clinics in the United States. To allow sufficient observational time, patients with less than one year of medical history in the EHR dataset before their COVID-19 tests were excluded (n = 42 365). Assessed COVID-19 outcomes including all-cause 30-day mortality, hospitalization, ICU admission, and ventilator use, which were compared using relative risks (RRs) according to cancer status and treatments.

**Results:**

Among 271 639 patients with COVID-19, 18 460 had at least one cancer diagnosis: 8034 with a history of cancer and 10 426 with newly diagnosed cancer within one year of COVID-19 infection. Patients with a cancer diagnosis were older and more likely to be male, white, Medicare beneficiaries, and have higher prevalences of chronic conditions. Cancer patients had higher risks for 30-day mortality (RR 1.07, 95% CI 1.01–1.14, P = 0.028) and hospitalization (RR 1.04, 95% CI 1.01–1.07, P = 0.006) but without significant differences in ICU admission and ventilator use compared to non-cancer patients. Recent cancer diagnoses were associated with higher risks for worse COVID-19 outcomes (RR for mortality 1.17, 95% CI 1.08–1.25, P<0.001 and RR for hospitalization 1.10, 95% CI 1.06–1.14, P<0.001), particularly among recent metastatic (stage IV), hematological, liver and lung cancers compared with the non-cancer group. Among COVID-19 patients with recent cancer diagnosis, mortality was associated with chemotherapy or radiation treatments within 3 months before COVID-19. Age, black patients, Medicare recipients, South geographic region, cardiovascular, diabetes, liver, and renal diseases were also associated with increased mortality.

**Conclusions and relevance:**

Individuals with cancer had higher risks for 30-day mortality and hospitalization after SARS-CoV-2 infection compared to patients without cancer. More specifically, patients with a cancer diagnosis within 1 year and those receiving active treatment were more vulnerable to worse COVID-19 outcomes.

## Introduction

Coronavirus disease (COVID-19) caused by severe acute respiratory syndrome coronavirus 2 (SARS-CoV-2) disproportionately affects individuals with underlying medical conditions [1,2]. Cancer has been considered a risk factor for severe COVID-19 outcomes [3]. Because of a compromised immune system due to cancer or cancer treatment, patients with cancer are generally more susceptible to infectious agents which may lead to increased morbidity and mortality risks from COVID-19. Earlier, generally smaller, studies reported that COVID-19 patients with cancer were at a higher risk for severe complications or death compared to those without cancer, especially individuals with lung cancers, hematological cancers, metastatic cancer, or recent cancer treatment [4–7]. However, more recent findings have been somewhat inconsistent. Some multicenter studies with a larger sample size of cancer patients, with matched or comparable non-cancer patients, confirmed that patients with cancer were at increased risk for COVID-19 infection and worse outcomes [8–10], while other studies claimed that cancer diagnosis or treatment was not associated with the outcomes [11–15]. Recent studies showed that the rates of severe illness from COVID-19 were comparable between those with and without cancer [13,14], and worse outcomes were driven by pre-existing conditions or initial COVID-19 severity, but not cancer characteristics [11,12,15].

Reported factors associated with increased mortality from COVID-19 in patients with cancer were similar to those without cancer, which include age, male sex, smoking, and the number of comorbidities [4–7,10]. Studies have also identified racial disparities related to COVID-19 outcomes in cancer patients. For example, non-Hispanic black and Hispanic patients were at higher risk for poorer COVID-19 outcomes in cancer patients [10,16,17].

Despite the accumulation of real-world data and evidence, the effect of cancer on COVID-19 outcomes has not been fully characterized. The impact of factors such as cancer treatment and social determinant remains to be elucidated. However, determining the independent contributing effect of cancer on COVID-19 outcomes is challenging because cancer and COVID-19 have many shared risk factors including older age and other comorbidities such as obesity. The goal of this study is to leverage the large-scale, Optum® de-identified COVID-19 Electronic Health Record (EHR) data set derived from more than 700 hospitals and 7000 clinics in the United States to systematically compare COVID-19 outcomes between patients with and without cancer. We are interested in studying questions such as whether recent diagnosis (within 1 year), active treatment, type of cancer, and other factors are associated with 30day mortality among COVID-19 patients with cancer after adjusting for other comorbidities. This large set of available cancer-related EHR data has also allowed us to perform subgroup analyses by comparing COVID-19 outcomes according to cancer type and cancer treatment and non-cancer counterparts, and to determine prognostic factors among COVID-19 patients with cancer. To account for the recognized overrepresentation of severe COVID-19 cases early in the pandemic, evidenced by substantially high hospitalization and death rates in early studies, this study excluded COVID-19 cases before June 2020 in analysis.

## Materials and methods

### Data source

This study uses licensed data from the Optum®. In response to the urgent need to understand the clinical impact of SARS-CoV-2 infection, Optum® developed a data pipeline with a minimal time lag while preserving as much clinical information as possible. The data is sourced and de-identified from Optum®’s longitudinal EHR repository derived from more than 700 hospitals and 7000 clinics in the United States. The COVID-19 dataset incorporates a wide swath of raw clinical information, including new, unmapped COVID-specific clinical data points from both Inpatient and Ambulatory electronic medical records. At the time of our study, the dataset includes patient-level, longitudinal clinical records including demographics, diagnoses, procedures, lab tests, care settings, medications prescribed or administered, and mortality for about 4.2 million unique individuals. The study protocol was reviewed and approved by the Committee for the Protection of Human Subjects (CPHS) at The University of Texas Health Science Center at Houston. Our study followed the Strengthening the Reporting of Observational Studies in Epidemiology (STROBE) reporting guideline.

### Study population

**Fig 1:**
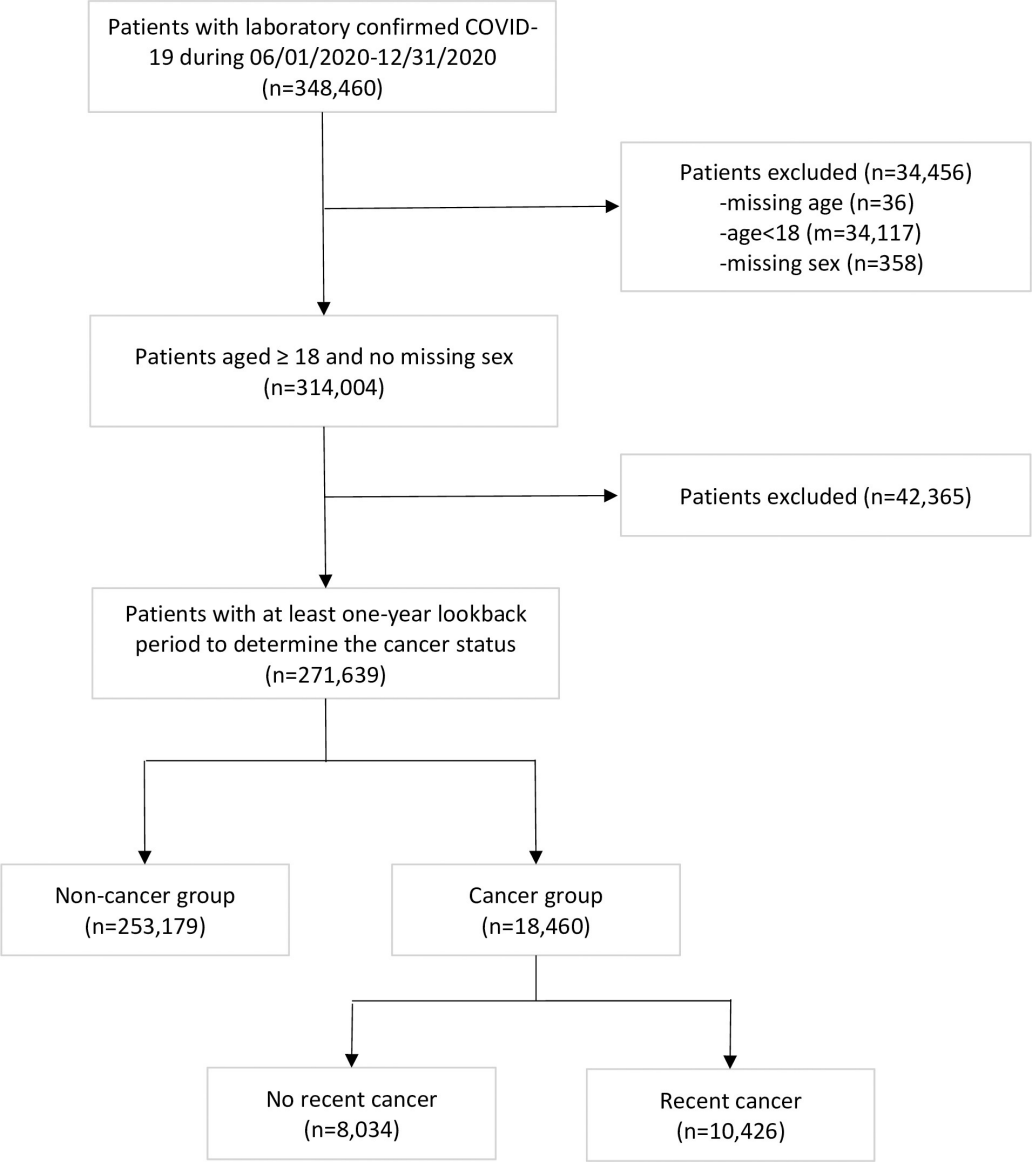
Cohort derivation.

Patients were included if they had laboratory-confirmed COVID-19 between June 1, 2020, and December 31, 2020, with a record of cancer diagnosis code before COVID-19 or without any evidence of cancer diagnosis in any time point (n = 348 460). To minimize potential changes in available meaningful treatments and mortality from infection with a new variant of SARS-CoV-2, we did not include either earlier infections before June 2020 or later infections on or after December 31, 2020 [18,19]. Positive COVID-19 status was determined by the detection of SARS-CoV-2 in the polymerase chain reaction (PCR) test, and the positivity date was based on the date of sample collection. Patients who were younger than 18 years (n = 34 117) or had missing age (n = 36) or missing sex information (n = 358) were excluded. To allow sufficient observational time to determine cancer status and baseline characteristics, patients with less than one year of medical history in the dataset prior to their COVID-19 tests were excluded (n = 42 365). The final cohort included 271 639 patients, 253 179 patients without any cancer history, and 18 460 patients with any cancer history (Fig 1).

### Cancer status and treatment

We determined the cancer status using the International Classification of Diseases, Ninth Revision, Clinical Modification (ICD-9-CM) and ICD-10-CM codes indicating any malignancy, including lymphoma and leukemia, except malignant nonmelanoma neoplasm of skin (S1 Table). Although ICD-9-CM codes have been replaced with ICD-10-CM as of October 2015, ICD-9 codes were included to identify cancer and comorbidities as some patient medical records were still reported using ICD-9 codes.

We conducted subgroup analyses limiting to patients with a recent history of cancer within one year before the COVID-19 date by the cancer type in reference to non-cancer patient subgroups including metastatic cancer, solid cancer, hematological cancer and most common 13 cancer types (S1 Table). Specific cancer stage information was not available but we determined metastatic cancer, that is commonly stage IV cancer, using ICD-9-CM and ICD-10-CM codes (S1 Table). Cancer treatments including chemo therapy and radiation therapy were identified using ICD-9-CM, ICD-10-CM, Berenson-Eggers Type of Service (BETOS), Current Procedural Terminology (CPT), Healthcare Common Procedure Coding System (HCPCS) (S2 Table) and National Drug Codes (NDCs) [20].

### Outcome measures and covariates

Our primary objective was to determine the effect of cancer on the outcomes of COVID-19, specifically hospitalization, intensive care unit (ICU) admission, ventilator use, and all-cause deaths occurring within 30 days of COVID-19 infection, by comparing these outcomes between COVID-19 patients with and without a cancer diagnosis. The secondary objective was to identify factors associated with mortality in COVID-19 patients with recent cancer treatment. Potential predictors included chemotherapy and radiation therapy within 3 months before the COVID-19, age, sex (not included for breast, endometrial, and prostate cancers), race/ethnicity, insurance type, regions, and comorbidity conditions such as chronic conditions identified and known risk factors (S3 Table) [21,22].

### Statistical analysis

Descriptive statistics for differences in baseline characteristics and outcome measures between cancer and non-cancer groups were assessed using the chi-square tests for categorical variables and Wilcoxon rank-sum tests for numeric variables. The effect of cancer status on outcomes was measured using the relative risk (RR) from a modified Poisson regression model including age, sex, race/ethnicity, insurance status, cancer treatment, and comorbidities as predictors [23]. To determine the effect of cancer type and stage, we measured RRs between each subgroup of cancer patients and non-cancer patients. Significance levels were set at P < 0.05 for 2-tailed tests and all analyses were performed using STATA 16.0 (StataCorp, College Station, TX).

## Results

### Characteristics of patients with and without cancer

The final cohort included 271 639 patients with confirmed COVID-19. We identified 18,460 patients with at least one cancer diagnosis and of them: 8034 patients with a history of cancer more than 1 year before COVID-19 and 10 426 patients with a recent cancer diagnosis, defined as cancer diagnosed within one year before COVID-19. Patients with a cancer diagnosis were older (Median age 66 [56–76] in cancer vs 46 [32–60] in non-cancer), more likely to be male (45% vs 43%), White (80% vs 72%), Medicare beneficiaries (33% vs 11%), and have higher prevalences of comorbidity conditions (Table 1). Baseline characteristics of patients among the cancer group were similar between cancer history and recent cancer groups (Table 1).

**Table 1:** Characteristics of patients in COVID-19 with or without cancer^a^.

			Of patients with cancer	
Characteristics	No cancer (n = 253,179)	Cancer (n = 18,460)	History of cancer (n = 8,034)	Recent cancer (n = 10,426)
**Age, yr, median [IQR]**	46 [32–60]	66 (56–76)	65 [55–77]	66 [57–76]
**Age group**				
18–44	119,419 (47.2)	1,837 (10.0)	934 (11.6)	903 (8.7)
45–54	45,383 (17.9)	2,354 (12.8)	1,071 (13.3)	1,283 (12.3)
55–64	43,791 (17.3)	4,423 (24.0)	1,870 (23.3)	2,553 (24.5)
65–74	25,151 (9.9)	4,556 (24.7)	1,815 (22.6)	2,741 (26.3)
75+	19,435 (7.7)	5,290 (28.7)	2,344 (29.2)	2,946 (28.3)
**Male**	108,203 (42.7)	8,354 (45.3)	3,424 (42.6)	4,930 (47.3)
**Race/Ethnicity**				
White	180,960 (71.5)	14,748 (79.9)	6,517 (81.1)	8,231 (78.9)
Black	25,644 (10.1)	1,719 (9.3)	696 (8.7)	1,023 (9.8)
Hispanic	26,262 (10.4)	1,221 (6.6)	470 (5.9)	751 (7.2)
Other/unknown	20,313 (8.0)	772 (4.2)	351 (4.4)	421 (4.0)
**Insurance**				
Commercial	165,986 (65.6)	9,926 (53.8)	4,404 (54.8)	5,522 (53.0)
Medicare	27,748 (11.0)	6,066 (32.9)	2,478 (30.8)	3,588 (34.4)
Medicaid	20,645 (8.2)	875 (4.7)	353 (4.4)	522 (5.0)
Uninsured	8,442 (3.3)	273 (1.5)	124 (1.5)	149 (1.4)
Other	12,078 (4.8)	662 (3.6)	294 (3.7)	368 (3.5)
Unknown	18,280 (7.2)	658 (3.6)	381 (4.7)	277 (2.7)
**Region**				
Northeast	36,021 (14.2)	3,407 (18.5)	1,465 (18.2)	1,942 (18.6)
Midwest	147,304 (58.2)	10,001 (54.2)	4,280 (53.3)	5,721 (54.9)
South	45,318 (17.9)	3,553 (19.2)	1,644 (20.5)	1,909 (18.3)
West	15,867 (6.3)	1,099 (6.0)	462 (5.8)	637 (6.1)
Other/Unknown	8,669 (3.4)	400 (2.2)	183 (2.3)	217 (2.1)
**Risk Factors**				
Chronic Pulmonary Disease	62,429 (24.7)	7,665 (41.5)	3,263 (40.6)	4,402 (42.2)
Cardiovascular Disease	49,725 (19.6)	8,883 (48.1)	3,700 (46.1)	5,183 (49.7)
Cerebrovascular Disease	15,599 (6.2)	3,475 (18.8)	1,489 (18.5)	1,986 (19.0)
Peripheral Vascular Disease	14,708 (5.8)	4,210 (22.8)	1,636 (20.4)	2,574 (24.7)
Diabetes	42,542 (16.8)	6,267 (33.9)	2,619 (32.6)	3,648 (35.0)
Obesity	80,978 (32.0)	8,383 (45.4)	3,596 (44.8)	4,787 (45.9)
Liver Disease	18,270 (7.2)	3,716 (20.1)	1,378 (17.2)	2,338 (22.4)
Renal Disease	18,145 (7.2)	4,518 (24.5)	1,843 (22.9)	2,675 (25.7)
**COVID-19 Outcomes**				
Death 30 days	4,854 (1.9)	1,258 (6.8)	477 (5.9)	781 (7.5)
Inpatient	25,092 (9.9)	4,060 (22.0)	1,584 (19.7)	2,476 (23.7)
ICU	6,449 (2.5)	1,101 (6.0)	427 (5.3)	674 (6.5)
Ventilator	2,855 (1.1)	510 (2.8)	178 (2.2)	332 (3.2)

Abbreviations: IQR, interquartile range; ICU, intensive care unit.

^a^Data are presented as number (percentage) of patients unless otherwise indicated. All p values for differences in characteristics between non-cancer and cancer groups are <0.001.

### COVID-19 outcomes with and without cancer

All-cause 30-day mortality was greater than three times in the cancer cohort compared to the non-cancer cohort (6.8% vs 1.9%), and the percentage of COVID-19 patients with cancer who required hospitalization, ICU admission, and ventilator use were more than twice times higher than in patients without cancer (Table 1). After adjusting for age, sex, race/ethnicity, and risk factors, we found that the cancer was associated with 7% increased mortality (RR 1.07, 95% CI 1.01–1.14, P = 0.028) and 4% increased hospitalization (RR 1.04, 95% CI 1.01–1.07, P = 0.006) (Tables 2 and S4). When we compared patients with a history of cancer to those without cancer, mortality and hospitalization were greater but not statistically significant. When we further compared patients with a history of cancer to those with recent cancer, the percentage of COVID-19 patients with recent cancer who required hospitalization, ICU admission, and ventilator use were significantly higher than in patients with a history of cancer (Table 1). Compared the recent cancer group to the non-cancer group, differences in mortality (RR 1.17, 95% CI 1.08–1.25, P<0.001) and hospitalization (RR 1.10, 95% CI 1.06–1.14, P<0.001) were greater. Subgroup analyses showed that recent metastatic and hematological cancers were associated with increased risk for worse outcomes compared to the non-cancer group (RRs of mortality 2.09, 95% CI 1.82–2.39, P<0.001 between recent metastatic vs non-cancer and 1.48, 95% CI 1.30–1.68, P<0.001 between recent hematological cancer vs non-cancer). Patients with recent solid cancer showed increased risk for mortality and hospitalization but not for ICU admission or ventilator use. Among specific cancer types, leukemia, liver, lung, and pancreatic cancers were associated with increased mortality (Table 2).

**Table 2:** COVID-19 outcomes by cancer status and types.

	Mortality ^a^		Hospitalization ^a^		ICU ^a^		Ventilator ^a^	
Cancer subtype (n)	ARR (95% CI)	p-value	ARR (95% CI)	p-value	ARR (95% CI)	p-value	ARR (95% CI)	p-value
None (253,179)	1.00 (Reference)		1.00 (Reference)		1.00 (Reference)		1.00 (Reference)	
Cancer (18,460)	1.07 (1.01–1.14)	0.028	1.04 (1.01–1.07)	0.006	0.97 (0.91–1.04)	0.41	0.95 (0.86–1.05)	0.29
History of cancer (8,034)	0.95 (0.87–1.03)	0.22	0.96 (0.92–1.01)	0.093	0.90 (0.82–0.99)	0.035	0.81 (0.70–0.94)	0.007
Recent cancer (10,426)	1.17 (1.08–1.25)	<0.001	1.10 (1.06–1.14)	<0.001	1.02 (0.95–1.11)	0.54	1.05 (0.93–1.17)	0.45
**Subgroup by recent cancer type**								
Metastatic (1580)	2.09 (1.82–2.39)	<0.001	1.26 (1.16–1.37)	<0.001	1.33 (1.13–1.56)	0.001	1.32 (1.04–1.68)	0.021
Solid (8952)	1.12 (1.04–1.22)	0.004	1.05 (1.00–1.09)	0.03	0.97 (0.89–1.06)	0.52	0.95 (0.84–1.08)	0.453
Hematological (2224)	1.48 (1.30–1.68)	<0.001	1.31 (1.22–1.40)	<0.001	1.27 (1.10–1.46)	0.001	1.41 (1.16–1.72)	0.001
**Cancer types**								
Bladder (476)	0.80 (0.62–1.05)	0.11	1.02 (0.88–1.17)	0.80	1.03 (0.77–1.36)	0.86	0.81 (0.51–1.27)	0.35
Breast† (2143)	1.08 (0.88–1.32)	0.47	0.99 (0.90–1.09)	0.876	0.92 (0.74–1.13)	0.409	1.06 (0.78–1.45)	0.70
Colorectal (794)	0.91 (0.69–1.19)	0.50	1.05 (0.93–1.20)	0.42	0.81 (0.61–1.07)	0.14	0.90 (0.61–1.35)	0.62
Endometrial (291)^b^	1.62 (0.96–2.74)	0.07	1.15 (0.90–1.46)	0.27	1.58 (1.03–2.41)	0.04	1.53 (0.81–2.90)	0.19
Kidney (474)	1.15 (0.86–1.53)	0.34	1.06 (0.91–1.22)	0.46	0.80 (0.57–1.13)	0.202	0.79 (0.49–1.30)	0.36
Leukemia (681)	1.58 (1.29–1.93)	<0.001	1.49 (1.34–1.66)	<0.001	1.38 (1.09–1.76)	0.008	1.73 (1.28–2.35)	<0.001
Liver (207)	2.46 (1.80–3.36)	<0.001	1.23 (1.00–1.51)	0.05	0.96 (0.62–1.49)	0.85	1.14 (0.63–2.05)	0.67
Lung (887)	1.85 (1.58–2.17)	<0.001	1.31 (1.19–1.44)	<0.001	1.35 (1.12–1.63)	0.002	1.21 (0.89–1.63)	0.22
Melanoma (409)	0.96 (0.67–1.38)	0.82	0.86 (0.69–1.06)	0.163	0.89 (0.57–1.38)	0.61	0.58 (0.27–1.27)	0.177
Non-Hodgkin Lymphoma (692)	1.02 (0.78–1.33)	0.89	1.23 (1.09–1.40)	0.001	1.02 (0.77–1.35)	0.90	1.49 (1.06–2.11)	0.02
Pancreatic (121)	1.94 (1.19–3.16)	0.008	0.98 (0.71–1.35)	0.91	0.72 (0.35–1.46)	0.363	0.84 (0.33–2.12)	0.71
Prostate (1781)^c^	0.82 (0.70–0.96)	0.015	0.95 (0.88–1.02)	0.177	0.81 (0.68–0.96)	0.017	0.78 (0.60–1.01)	0.055
Thyroid (476)	0.83 (0.46–1.51)	0.55	0.76 (0.59–0.98)	0.035	0.58 (0.30–1.11)	0.10	0.38 (0.12–1.19)	0.10

^a^Adjusted relative risk (ARR) were estimated from modified multivariable Poisson regressions in reference to non-cancer group. All RRs were adjusted for age-group, sex (except breast, endometrial and prostate cancers), race/ethnicity, insurance type, regions, chronic pulmonary, cardiovascular, cerebrovascular, peripheral vascular, diabetes, obesity, liver, and renal diseases.

^b^Analysis was limited to female only.

^c^Analysis was limited to male only.

### Factors associated with the mortality in COVID-19 with recent cancer

Table 3 shows factors associated with the 30-day mortality among COVID-19 patients with recent cancer. Recent cancer patients who were undergoing active treatment within 3 months prior to the COVID-19 were more likely to die (RR 1.37, 95% CI 1.12–1.69, P = 0.003 with chemo and RR 1.83, 95% CI 1.37–2.46, P<0.001 with radiation) adjusting for age, sex, race/ethnicity, insurance type, region, and risk factors. Old age increased the risk of death substantially (RRs 6.29, 95% CI 3.35–11.83, P<0.001 between age 65–74 and <45 and 14.41, 95% CI 7.73–26.83, P<0.001) between 75+ and <45) but male sex was not significantly associated with the mortality after adjusting for covariates. Black race (RR 1.25, 95% C 1.02–1.53, P = 0.029), Medicare beneficiaries (RR 1.29, 95% CI 1.11–1.51, P = 0.001) and South region (RR 1.64, 95% CI 1.33–2.03, P<0.001) were independently associated with the increased risk of death. Among known risk factors, cardiovascular disease, diabetes, liver and renal diseases were significantly associated with the increased risk of death.

**Table 3:** Factors associated with the mortality in COVID-19 with recent cancer.

	Crude RR	p-value	adjusted RR	p-value
**Cancer Treatment**				
Chemo within 3-month before COVID-19	1.31 (1.08–1.60)	0.006	1.37 (1.12–1.69)	0.003
Radiation within 3-month before COVID-19	1.89 (1.44–2.49)	<0.001	1.83 (1.37–2.46)	<0.001
**Age group**				
<45	1 (reference)			
45–54	1.41 (0.66–2.99)	0.37	1.21 (0.57–2.57)	0.62
55–64	3.18 (1.66–6.09)	<0.001	2.28 (1.19–4.37)	0.013
65–74	6.29 (3.35–11.83)	<0.001	3.50 (1.83–6.69)	<0.001
75+	14.41 (7.73–26.83)	<0.001	6.69 (3.50–12.78)	<0.001
**Male**	1.63 (1.47–1.82)	<0.001	1.12 (0.98–1.28)	0.106
**Race/ethnicity**				
White	1 (reference)			
Black	1.26 (1.03–1.54)	0.027	1.25 (1.02–1.53)	0.029
Hispanic	0.67 (0.49–0.92)	0.014	0.93 (0.68–1.28)	0.65
Other/unknown	0.82 (0.56–1.20)	0.31	1.08 (0.74–1.58)	0.704
**Insurance Type**				
Commercial	1 (reference)			
Medicare	2.37 (2.05–2.74)	<0.001	1.29 (1.11–1.51)	0.001
Medicaid	0.87 (0.57–1.32)	0.51	1.37 (0.89–2.10)	0.154
Uninsured	0.53 (0.20–1.40)	0.20	0.73 (0.28–1.88)	0.51
Other	1.45 (0.99–2.12)	0.057	1.40 (0.98–2.01)	0.066
Unknown	1.14 (0.70–1.86)	0.602	1.24 (0.77–2.00)	0.38
**Region**				
Northeast	1 (reference)			
Midwest	1.03 (0.85–1.26)	0.73	1.07 (0.88–1.30)	0.507
South	1.70 (1.38–2.10)	<0.001	1.64 (1.33–2.03)	<0.001
West	1.17 (0.85–1.61)	0.335	1.22 (0.89–1.68)	0.208
Other/Unknown	1.29 (0.80–2.07)	0.294	1.19 (0.76–1.88)	0.444
**Risk Factors**				
Chronic Pulmonary Disease	1.42 (1.24–1.63)	<0.001	0.96 (0.84–1.10)	0.56
Cardiovascular Disease	3.50 (2.97–4.13)	<0.001	1.72 (1.43–2.07)	<0.001
Cerebrovascular Disease	1.98 (1.71–2.28)	<0.001	1.09 (0.94–1.26)	0.28
Peripheral Vascular Disease	2.01 (1.75–2.30)	<0.001	1.03 (0.89–1.19)	0.69
Diabetes	2.04 (1.79–2.34)	<0.001	1.39 (1.20–1.60)	<0.001
Obesity	0.96 (0.84–1.10)	0.571	0.86 (0.75–0.99)	0.039
Liver Disease	1.23 (1.06–1.43)	0.008	1.16 (1.00–1.36)	0.05
Renal Disease	2.80 (2.45–3.20)	<0.001	1.51 (1.31–1.75)	<0.001

## Discussion

We found that cancer diagnosis was in general associated with an increased risk for mortality and hospitalization among COVID-19 patients. However, a history of cancer more than one year before COVID-19 diagnosis was not significantly associated with increased mortality or hospitalization. Recent cancer, particularly recent metastatic (stage IV), hematological, liver, and lung cancers were associated with worse COVID-19 outcomes compared to the non-cancer group. Higher mortality rate was associated with active radiation or systemic therapy within 3 months before COVID-19 diagnosis. Older age, black race, Medicare recipients, South geographic region, cardiovascular, diabetes, liver, and renal diseases were also independently associated with increased risks for 30-day mortality of COVID-19.

Our findings were consistent with earlier studies and some of the recent studies [4–10] on the association between cancer-specific factors and increased mortality. However, we found mortality and hospitalization rates to be significantly lower in both cancer and non-cancer groups compared to those reported in other studies. For example, in a similar study using multi-centered EHR data, Wang et al reported 14.9% death rate among cancer COVID-19 patients and 5.3% among non-cancer patients [10]. Considering the fact that the estimated mortality rates are less than 2% among the general population, such a rate for non-cancer patients appear to be inflated [24]. In contrast, our study found 6.8% mortality in the cancer group and 1.9% mortality in the non-cancer group, closer to reported rates within the general population. Caution must be exercised in treating early pandemic period data, as we did, when (prior June 2020) comprehensive testing was not widely available and treatment strategies were still been identified, which could result in an overrepresentation of severe COVID-19 outcomes.

Our results also confirmed racial disparities in COVID-19 treatment and outcomes among COVID-19 patients with cancer [10,16,17,25]. Among hospitalized COVID-19 patients with cancer, the administration of remdesivir was 45.8% but it was lower among black patients than white patients (39.3% vs 47.7%, P<0.001). The 30-day mortality rate among cancer patients was higher among black patients (9.5%) compared to white patients (7.5%) or Hispanic patients (5.1%). After adjusting for covariates, black race remained to be associated with a higher risk for death (RR 1.25, 95% CI 1.02–1.53, P = 0.029).

Contrary to our findings, some recent studies have shown that rates of severe illness from COVID-19 were comparable between those with and without cancer and reported no effect of cancer characteristics on COVID-19 outcomes [11–15]. A study of 928 patients from the United States, Canada, and Spain enrolled in the COVID-19 and Cancer Consortium reported no increased risk of death associated with cancer type or timing of cancer treatment [26]. Another analysis of 423 patients with symptomatic COVID-19 at a New York cancer center found that neither recent receipt of chemotherapy or surgery nor having metastatic cancer were associated with higher risks of complications [27]. These studies have reported higher risks of worse outcomes for patients with a cancer diagnosis overall but adjusting for pre-existing conditions resulted in negation of these worse outcomes [11,12,15]. It is possible that in these studies, the number of cancer patients may have been too small to find statistical significance when adjusting for demographics, geolocation, and multiple comorbidities. Our study had a substantial number of patients in subgroups by cancer types, which allowed us to compare COVID-19 outcomes for each cancer type with matched non-cancer patients. In addition to hematological and lung cancer, which have been previously reported to be associated with worse outcomes, we also found that liver and pancreatic cancers were associated with increased mortality (RR 2.46, 95% CI 1.80–3.36, P<0.001 for liver cancer vs non-cancer, RR 1,94, 95% CI 1.19–3.16, P = 0.008 for pancreatic cancer vs non-cancer).

Our study has several limitations. First, not specifically collected for oncology, the source data had limited resolution for certain cancer characteristics. We relied on ICD-9 and 10 codes to identify cancer patients and had limited cancer staging information except the determination of stage IV cancer (metastatic). Second, we defined the diagnosis of cancer based on the occurrence of diagnosis codes, which could result in false positive classification of cancer diagnosis. However, despite the potential false positive identification of cancer history, we still observed worse outcomes for patients with a cancer diagnosis compared with patients without cancer. To account for potential missing or misclassifications of cancer cases, comorbidities, and cancer treatments, we required a lookback period as an inclusion criterion and used combinations of codes to capture chemotherapy and radiation treatments to minimize misclassification errors.

## Conclusion

In this large-scale population study confirmed an increased risk of death and hospitalization among COVID-19 patients with any diagnosis of cancer compared to non-cancer patients. However, individuals with a cancer diagnosis within 1 year of COVID-19 and those who had received cancer treatment within 3 months of COVID-19 were associated with notably worse COVID-19 outcomes. Among cancer patients, a diagnosis of hematological malignancies, lung, liver and pancreatic cancer were independently associated with worse outcomes. Finally, this study reaffirmed the presence of racial disparities in COVID-19 treatment and outcomes among particularly vulnerable populations.

## Supporting information

S1 Table Identification of Cancer using the International Classification of Diseases, Ninth Revision, Clinical Modification (ICD-9-CM) and ICD-10-CM codes. (DOCX)

S2 Table Codes to identify cancer treatments. (DOCX)

S3 Table Codes to identify comorbidities. (DOCX)

S4 Table Factors associated with COVID-19 outcomes in patients with/without cancer. (DOCX)
