# Supplementary material for: Characterizing cancer and COVID-19 outcomes using electronic health records
Source: PLoS One. 2022 May 4;17(5):e0267584. doi: 10.1371/journal.pone.0267584 (PMC9067885; doi:10.1371/journal.pone.0267584)
Supplement: S1 Table — (DOCX) [file pone.0267584.s001.docx]

**S1 Table.** Identification of Cancer using the International Classification of Diseases, Ninth Revision, Clinical Modification (ICD-9-CM) and ICD-10-CM Codes

|  | **ICD-9-CM** | **ICD-10-CM** |
| --- | --- | --- |
| **Cancer Status** |  |  |
| Any malignancy, including lymphoma and leukemia, except malignant nonmelanoma neoplasm of skin | 14x.x, 15x.x, 16x.x, 170.x-172.x, 174.x-176.x, 179.x, 18x.x, 190.x- 208.x, 209.0x-209.3x, 209.7x | C0x.x, -C3x.x, C40.x, C41.x, C43.x C45.x-C49.x, C5x.x, C6x.x, C7x.x, C8x.x, C9x.x |
| **Cancer Types** |  |  |
| Solid Tumors | 14x,15x,16x,17x,18x,19x | C0, C1, C2, C3, C4, C5, C6, C7 |
| Hematological malignancies | 20x.x | C8, C9 |
| **13 Common Cancers** |  |  |
| Bladder | 188.x | C67.x |
| Breast | 174.x | C50.x |
| Colorectal | 153.x, 154.0x, 154.1x, 154.8x | C18.x, C19.x, C20.x, C21.8x |
| Endometrial | 182.x | C54.x |
| Kidney (Renal Cell and Renal Pelvis) | 189.0x, 189.1x | C64.x, C65.x |
| Leukemia (All Types) | 204.x, 205.x, 206.x, 207.x, 208.x | C91.x, C92.x, C93.x, C94.x, C95.x |
| Liver (Liver, Gallbladder, and Biliary Tract) | 155.x, 156.x | C22.x, C23.x, C24.x |
| Lung | 162.x | C34.x |
| Melanoma | 172.x | C43.x |
| Non-Hodgkin Lymphoma | 200.x, 202.x | C82.x, C83.x, C84.x, C85.x, C86.x, C88.x |
| Pancreatic | 157.x | C25.x |
| Prostate | 185.x | C61.x |
| Thyroid | 193.x | C73.x |
| **Cancer Stage** |  |  |
| Metastatic (Stage IV) | 196.x, 197.x, 198.x | C7B, C77, C78, C79 |
