# Supplementary material for: Characterizing cancer and COVID-19 outcomes using electronic health records
Source: PLoS One. 2022 May 4;17(5):e0267584. doi: 10.1371/journal.pone.0267584 (PMC9067885; doi:10.1371/journal.pone.0267584)
Supplement: S2 Table — (DOCX) [file pone.0267584.s002.docx]

**S2 Table.** Codes to Identify Cancer Treatments

| **Treatment** | **Codes** |
| --- | --- |
| **Chemotherapy** | **BETOS**: O1D |
|  | **ICD-9-CM Diagnosis**: V58.11 |
|  | **ICD-10-CM**: Z51.11 |
|  | **CPT/HCPCS**: 96400-96549, Q0083-Q0085, 51720 (bladder instillation) |
|  | **HCPCS for Specific drugs** |
|  | C9065, C9253, C9474, C9480, J0594, J0894, J3305, J7527, J8510, J8520, |
|  | J8521, J8530, J8560, J8565, J8600, J8610, J8700, J8705, J8999, J9000, |
|  | J9017, J9019, J9020, J9025, J9027, J9032, J9033, J9034, J9036, J9040, |
|  | J9041, J9043, J9045, J9047, J9050, J9060, J9065, J9070, J9092, J9094, |
|  | J9097, J9098, J9100, J9118, J9120, J9130, J9150, J9151, J9171, J9178, |
|  | J9179, J9181, J9185, J9190, J9198, J9200, J9201, J9206, J9207, J9208, |
|  | J9211, J9223, J9230, J9245, J9246, J9250, J9260, J9261, J9262, J9263, |
|  | J9264, J9266, J9267, J9268, J9270, J9280, J9281, J9293, J9304, J9305, |
|  | J9307, J9315, J9320, J9328, J9330, J9340, J9351, J9357, J9360, J9370, |
|  | J9371, J9390, J9400, J9600, J9999, Q2017, Q2049, Q2050, S0088, S0108, |
|  | S0172, S0176, S0178, S0182 |
|  | **Revenue Codes**: 0331, 0332, 0335 |
| **Radiation therapy** | **BETOS**: P7A |
|  | **ICD-9-CM Diagnosis**: V58.0 |
|  | **ICD-10-CM**: Z51.0 |
|  | **CPT/HCPCS**: 77401-77499, 77520, 77523, 77750-77799, G0256, G0261 |
|  | **Revenue Codes**: 0330, 0333 |

Abbreviations: BETOS, Berenson-Eggers Type of Service; ICD, International Classification of Diseases; CPT, Current Procedural Terminology; HCPCS, Healthcare Common Procedure Coding System.
