# Supplementary material for: Characterizing cancer and COVID-19 outcomes using electronic health records
Source: PLoS One. 2022 May 4;17(5):e0267584. doi: 10.1371/journal.pone.0267584 (PMC9067885; doi:10.1371/journal.pone.0267584)
Supplement: S3 Table — (DOCX) [file pone.0267584.s003.docx]

**S3 Table.** Codes to Identify Comorbidities

| **Comorbidities** | **ICD-9-CM** | **ICD-10-CM** |
| --- | --- | --- |
| Chronic pulmonary disease | 416.8, 416.9, 490.x - 505.x, 506.4, 508.1, 508.8 | I27.8x, I27.9, J40.x - J47.x, J60.x - J67.x, J68.4, J70.1, J70.3 |
| Cardiovascular Disease | 410.x, 411.x, 412.x, 413.x, 414.x, 427.x, 428.x | I20.x, I21.x, I22.x, I23.x, I25.x, I46.x, I47.x, I48.x, I49.x, I50.x |
| Cerebrovascular disease | 362.34, 430.x–438.x | H34.0x, G45.x, G46.x, I60.x - I69.x |
| Peripheral vascular disease | 093.0, 440.x, 441.x, 443.1, 443.2x, 443.8x, 443.9, 447.1, 557.1, 557.9, V43.4 | A52.01, I70.x, I71.x, I73.1, I73.8x, I73.9, I77.1, I79.x, K55.1, K55.8, K55.9, Z95.82x |
| Diabetes | 250.x | E10.x, E11.x, E13.x |
| Obesity | 278.0x | E66.x |
| Liver disease | 070.22, 070.23, 070.32, 070.33, 070.44, 070.54, 070.6, 070.9, 456.0, 456.1, 456.2x, 570.x, 571.x, 572.2, 572.3, 572.4, 572.8, 573.3, 573.4, 573.8, 573.9, V42.7 | B18.x, K70.0, K70.1x, K70.2, K70.3x, K70.4x, K70.9, K71.1x, K71.3, K71.4, K71.5x, K71.7, K72.1x, K72.9x, K73.x, K74.x, K76.x, Z94.4, I85.0x, I86.4 |
| Renal disease | 403.x, 404.x, 582.x, 583.0 - 583.7, 585.x, 586.x, 588.0, V42.0, V45.11, V56.x | I12.x, I13.x, N03.x, N05.2-N05.7, N18.x, N19.x, N25.0, Z94.0, Z99.2, Z49.x |
