# Supplementary material for: Characterizing cancer and COVID-19 outcomes using electronic health records
Source: PLoS One. 2022 May 4;17(5):e0267584. doi: 10.1371/journal.pone.0267584 (PMC9067885; doi:10.1371/journal.pone.0267584)
Supplement: S4 Table — (DOCX) [file pone.0267584.s004.docx]

**S4 Table.** Factors associated with COVID-19 outcomes in patients with/without cancer

|  | adjusted RR for Mortality | p-value | adjusted RR for Hospitalization | p-value |
| --- | --- | --- | --- | --- |
| **Cancer** | 1.07 (1.01 - 1.14) | 0.028 | 1.04 (1.01 - 1.07) | 0.006 |
| **Age group** |  |  |  |  |
| <45 | 1 (reference) |  |  |  |
| 45-54 | 3.99 (3.20 - 4.98) | <0.001 | 1.54 (1.48 - 1.60) | <0.001 |
| 55-64 | 10.07 (8.29 - 12.23) | <0.001 | 2.25 (2.17 - 2.34) | <0.001 |
| 65-74 | 22.33 (18.38 - 27.12) | <0.001 | 3.38 (3.24 - 3.52) | <0.001 |
| 75+ | 58.91 (48.44 - 71.64) | <0.001 | 4.93 (4.72 - 5.14) | <0.001 |
| **Male** | 1.34 (1.28 - 1.41) | <0.001 | 1.17 (1.15 - 1.19) | <0.001 |
| **Race/ethnicity** |  |  |  |  |
| White | 1 (reference) |  |  |  |
| Black | 1.18 (1.09 - 1.28) | <0.001 | 1.79 (1.74 - 1.85) | <0.001 |
| Hispanic | 1.12 (1.02 - 1.24) | 0.021 | 1.70 (1.65 - 1.76) | <0.001 |
| Other/unknown | 1.27 (1.15 - 1.40) | <0.001 | 1.45 (1.39 - 1.51) | <0.001 |
| **Insurance Type** |  |  |  |  |
| Commercial | 1 (reference) |  |  |  |
| Medicare | 1.32 (1.25 - 1.40) | <0.001 | 1.15 (1.12 - 1.19) | <0.001 |
| Medicaid | 1.62 (1.40 - 1.89) | <0.001 | 1.55 (1.49 - 1.62) | <0.001 |
| Uninsured | 1.40 (1.12 - 1.74) | <0.001 | 1.20 (1.13 - 1.29) | <0.001 |
| Other | 1.37 (1.20 - 1.56) | <0.001 | 1.10 (1.05 - 1.16) | <0.001 |
| Unknown | 1.45 (1.29 - 1.63) | <0.001 | 0.56 (0.52 - 0.59) | <0.001 |
| **Region** |  |  |  |  |
| Northeast | 1 (reference) |  |  |  |
| Midwest | 1.00 (0.92 - 1.07) | 0.92 | 0.66 (0.64 - 0.68) | <0.001 |
| South | 1.54 (1.43 - 1.67) | <0.001 | 1.19 (1.16 - 1.23) | <0.001 |
| West | 1.19 (1.07 - 1.33) | 0.002 | 1.08 (1.04 - 1.13) | <0.001 |
| Other/Unknown | 1.35 (1.16 - 1.58) | <0.001 | 0.87 (0.81 - 0.93) | <0.001 |
| **Risk Factors** |  |  |  |  |
| Chronic Pulmonary Disease | 1.08 (1.02 - 1.13) | 0.004 | 1.12 (1.09 - 1.14) | <0.001 |
| Cardiovascular Disease | 1.97 (1.84 - 2.10) | <0.001 | 1.38 (1.35 - 1.42) | <0.001 |
| Cerebrovascular Disease | 1.19 (1.13 - 1.26) | <0.001 | 1.05 (1.02 - 1.08) | <0.001 |
| Peripheral Vascular Disease | 0.98 (0.92 - 1.03) | 0.398 | 1.01 (0.98 - 1.04) | 0.43 |
| Diabetes | 1.30 (1.24 - 1.37) | <0.001 | 1.47 (1.43 - 1.50) | <0.001 |
| Obesity | 0.88 (0.84 - 0.93) | <0.001 | 1.20 (1.17 - 1.23) | <0.001 |
| Liver Disease | 1.01 (0.94 - 1.08) | 0.819 | 1.08 (1.05 - 1.11) | <0.001 |
| Renal Disease | 1.69 (1.60 - 1.79) | <0.001 | 1.27 (1.23 - 1.30) | <0.001 |
